# Biomechanical comparison of the bone‐screw‐fastener to conventional cortical buttress screw in a simulated ex vivo model of equine midbody proximal sesamoid bone fracture repair

**DOI:** 10.1111/vsu.70060

**Published:** 2025-11-25

**Authors:** Thomas J. O'Brien, James W. Johnson, Christopher E. Kawcak, Ben C. Gadomski, Ryan S. Carpenter, Brad B. Nelson

**Affiliations:** ^1^ Orthopedic Research Center, C. Wayne McIlwraith Translational Medicine Institute, Department of Clinical Sciences College of Veterinary Medicine and Biomedical Sciences Colorado State University Fort Collins Colorado USA; ^2^ Department of Mechanical Engineering, Orthopedic Bioengineering Research Laboratory Colorado State University Fort Collins Colorado USA; ^3^ Equine Medical Center Cypress California USA

## Abstract

**Objective:**

To compare implant failure and gap displacement characteristics of simulated medial mid‐body proximal sesamoid bone (PSB) fractures repaired with bone‐screw‐fasteners (BSF) or cortical screws (CS) in single or double screw configurations.

**Study design:**

Ex vivo experimental study.

**Sample population:**

A total of 14 paired equine cadaver forelimbs.

**Methods:**

Medial mid‐body PSB osteotomies were created in each forelimb. Surgical repair was performed using either: (1) single 3.5 mm BSF (BSF_single_), (2) single 4.5 mm CS (CS_single_), (3) two 3.5 mm BSFs (BSF_double_), or (4) two 3.5 mm cortical screws (CS_double_) (*n* = 7 repairs/group). Biomechanical properties and failure characteristics were evaluated through a single cycle to failure. Comparisons between groups were made using Wilcoxon‐matched pairs or Mann–Whitney tests. Statistical significance was *p* < .05.

**Results:**

The BSF_double_ group (2081 ± 181 N) had significantly higher yield than BSF_single_ (1458 ± 92 N, *p* = .01) and CS_single_ (1532 ± 86 N, *p* = .02) groups. The CS_double_ group (2101 ± 126 N) had significantly higher yield than BSF_single_ (*p* = .001) and CS_single_ (*p* = .003) groups. Biomechanical properties were not different between BSF_single_ and CS_single_ groups, or between BSF_double_ and CS_double_ groups. Gap measurements at construct failure were significantly higher abaxially than axially in all groups (all *p* < .05).

**Conclusion:**

No differences were detected between the single BSF and 4.5 mm CS for repair of medial mid‐body PSB fractures. Surgical repair using two screws has biomechanical advantage to single screw repair, regardless of screw type ex vivo.

**Clinical significance:**

The BSF is not different to CS for repair of PSB fractures. Counteracting abaxial forces in surgical repair of mid‐body PSB fractures warrants further investigation.

## 1 INTRODUCTION

Musculoskeletal injuries in racehorses represent significant morbidity and mortality and account for a high proportion of fatalities in racing Thoroughbreds and Quarter Horses.^1^ The proximal sesamoid bone (PSB) is a common site of fracture (30%–62%) occurring during racing or training typically resulting from repetitive cyclical PSB fatigue or possibly from monotonic overstrain injury.^1^, ^2^, ^3^, ^4^ Regardless of inciting cause, mid‐body PSB fractures separate the bone into similarly sized fragments. Limited repair options exist due to low bone availability and high loads applied to the PSB. Conservative management results in an unfavorable prognosis as tension from the suspensory apparatus leads to a weak fibrous union that causes inferior healing with ongoing instability and permanent lameness.^5^, ^6^ Arthroscopic removal of smaller fragments has favorable outcomes; however, removal of a mid‐body fracture fragment results in considerable disruption to the suspensory apparatus.^7^ Surgical repair of mid‐body PSB fractures using lag screw technique is recommended to optimize bone healing, though numerous complications can occur including implant breakage, palmar digital neuroma, fibrous union, persistent instability or basilar fracture.^5^, ^8^, ^9^ Breakdown injuries involving mid‐body fractures of both PSBs require fetlock arthrodesis as repair with screws are insufficient to counteract tension of the disrupted suspensory apparatus during weight bearing.^10^ Investigations of new implant designs are useful to improve and optimize PSB fracture repair.

Several studies have investigated and failed to identify an improved implant to repair mid‐body PSB fractures (headless tapered variable pitch screw, ultra‐high‐molecular weight polyethylene cable, stainless steel wire) compared to a single 4.5 mm cortical screw (CS).^11^, ^12^, ^13^ The current standard of care is surgical repair with either a single 4.5 mm or two 3.5 mm CSs.^10^ Failure often occurs at the fracture site whereby the proximal fragment is displaced when tension from the suspensory ligament exceeds the screw holding capacity preventing bony union.^11^, ^13^, ^14^ Failure after conventional lag screw repair may be due to the intrinsic limitations of the buttress thread configuration in CSs, which only resist unidirectional axial loading.^15^ Multidirectional forces are applied to the PSB (e.g., tension/compression, external rotation), risking the loosening and failure of the conventional cortical screw through repetitive loading during healing.^15^, ^16^ Further, no biomechanical studies have previously investigated two 3.5 mm screws for PSB fracture repair, despite its clinical use.

**FIGURE 1:**
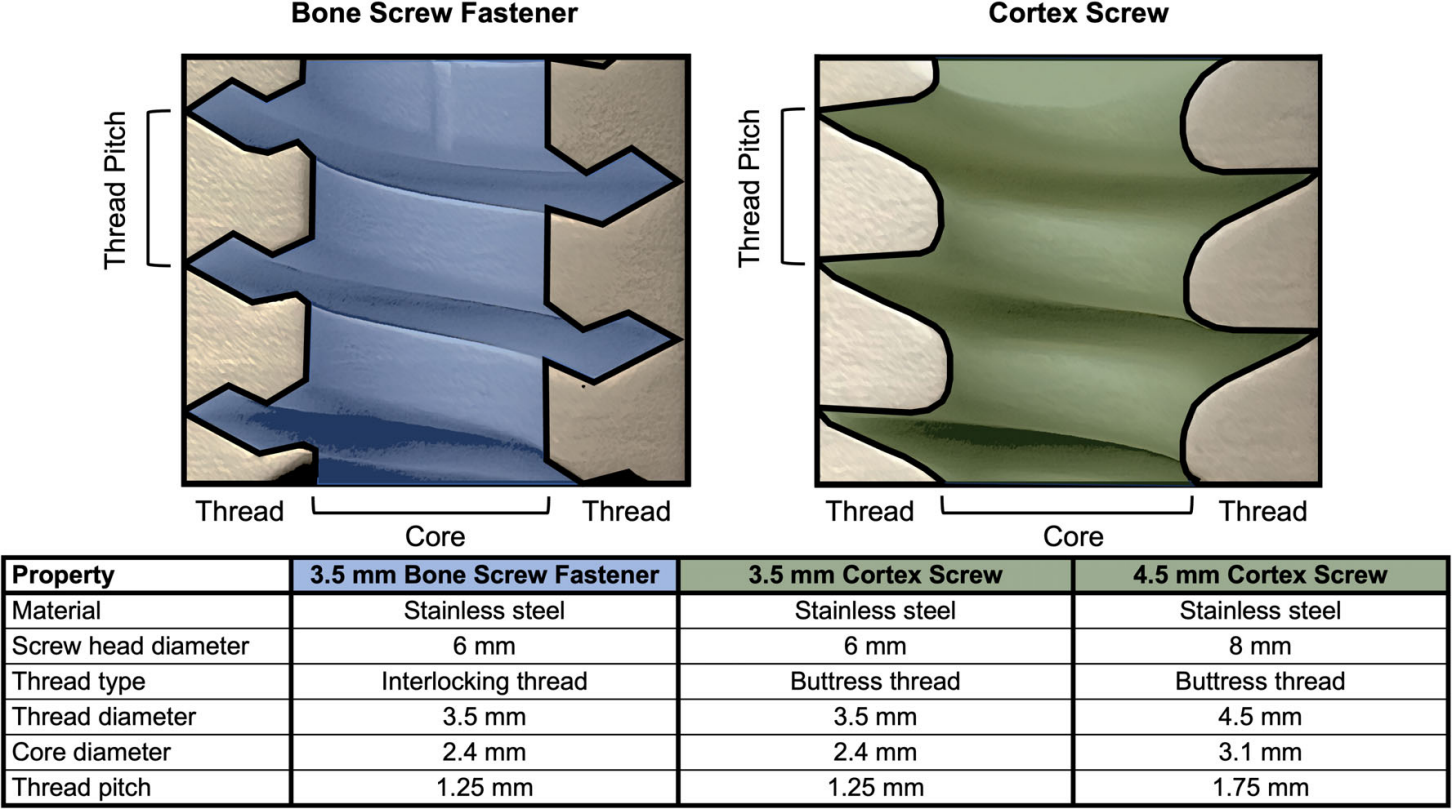
Negative thread hole impressions of simulated bone interdigitating with a bone screw fastener (left) or cortex screw (right). Screws are not shown. The bone screw fastener enables multidirectional compensation during eccentric loading compared to the cortex screw. Structural properties of the different screw types used to repair simulated ex vivo midbody medial proximal sesamoid bone fractures are detailed.

The bone‐screw‐fastener (BSF, OsteoCentric Technologies, Austin, Texas) is an implant with a unique interlocking thread design allowing engagement of both cis and trans cortices with an ability to resist multidirectional forces (Figure 1).^15^ Additionally, its interlocking thread design ultimately allows improved resistance to torque failure which corresponds to a higher stripping strength when compared to a conventional CS.^17^, ^18^, ^19^, ^20^ Study objectives were to compare four methods of surgical repair of medial mid‐body PSB fractures by evaluating biomechanical properties in a single cycle axial loading to failure. The four repair methods were: (1) single 3.5 mm BSF (BSF_single_), (2) single 4.5 mm CS (CS_single_), (3) two 3.5 mm BSFs (BSF_double_), or (4) two 3.5 mm CSs (CS_double_). The biomechanical properties measured included loaded gap displacement, yield failure and construct failure. We hypothesized that BSF_single_ would have reduced gap formation at the fracture site and a higher yield to failure compared to CS_single_, and that double screw repair (CS_double_ or BSF_double_) would have higher yield failure and reduced gap **formation compared to single screw repair (BSF**_**single**_ **or CS**_**single**_).

## 2 MATERIALS AND METHODS

### 2.1 Study design

A total of 28 forelimbs were collected from 14 horses donated to the Johnson Family Equine Hospital at Colorado State University after euthanasia for problems unrelated to the musculoskeletal system. Client consent for the use of cadaveric specimens was obtained. Horse breeds included Quarter Horse (*n* = 4), Thoroughbred (*n* = 4), Warmblood (*n* = 3) and other (*n* = 3). There were 10 geldings and four mares with mean ± SD ages of 9.8 ± 4.4 years. Gross and radiographic evaluation of the metacarpophalangeal joint confirmed all limbs had no visible abnormalities of the metacarpophalangeal joint, suspensory apparatus or PSBs. Within 24 h following euthanasia, limbs were disarticulated through the middle carpal joint. The skin, superficial and deep digital flexor tendons were removed. The suspensory apparatus was kept intact and examined grossly for any evidence of injury. Any visible injuries would have excluded the limbs from use. Limbs were wrapped in 0.9% saline‐soaked gauze and stored at −20°C.

Limbs were thawed at room temperature (27°C) for 12 h prior to PSB osteotomy and surgical repair. A transverse mid‐body osteotomy was created in each medial PSB while maintaining an intact suspensory apparatus. One limb from each horse was repaired with one of the BSF groups, while the other was repaired with one of the CS groups. In the first seven horses, limbs were repaired with a single screw, either BSF_single_ or CS_single_. The next seven paired limbs were repaired with two screws, either BSF_double_ or CS_double_. Specific implant characteristics of the BSF are published by Pye et al.^17^

### 2.2 Surgical repair

All surgical repairs were performed by the same equine surgery resident (TJO) with supervision by an ACVS board‐certified surgeon (BBN). Each forelimb was placed on a table. The apex, mid‐body, and base of each medial PSB were marked using 18‐gauge, 3.81 cm needles and positioning verified using fluoroscopy. The osteotomy site was marked using a ruler and permanent marker from the abaxial to axial portion across the palmar fibrocartilage, halfway between the base and apex of the PSB. A transverse osteotomy was created using an oscillating saw (Arthrex: AR‐300‐043S, 0.55 mm thickness), with constant 0.9% saline irrigation extending through the palmar fibrocartilage. Fluoroscopy images with distraction of the apical and basilar segments with fetlock extension were used to complete PSB transection. A stab incision was made at the base of the medial PSB using a No.15 scalpel blade at the planned screw insertion site of each limb. Single and double screw insertions were performed in the following sequence: glide hole drilling, PSB fracture reduction, thread hold drilling, countersinking preparation, screw length measurement, thread hole tapping and screw placement.

For the BSF_single_ group, a 3.5 mm glide hole was created on a trajectory from the mid‐point of the base at the natural fossa towards the apex. Drill trajectory was determined via fluoroscopy. The glide hole was confirmed to enter the fracture gap. A 3.5 mm drill sleeve was inserted and pointed bone reduction forceps were tightened to reduce the fracture. Even alignment (visually) of the palmar fibrocartilage and of the PSB in dorsopalmar and lateromedial fluoroscopic projections images confirmed fracture reduction. The 2.5 mm thread hole was drilled into the proximal fragment. Countersinking was performed using the dedicated countersink instrument (4.5 mm or 3.5 mm) within each set for each screw size. Approximately 3–4 turns were used to lightly remove bone at the PSB base to improve the bone‐screw head contact and was performed identically regardless of screw type. Screw length was measured with a depth gauge, and inserted screws were confirmed on fluoroscopy to have threads fully engaging the PSB apex. Tapping of the hole was performed manually using a specific BSF tap to accommodate the interlocking threads (OsteoCentric). Tactile sensation of smooth tap rotation along with periodic fluoroscopy images were taken to ensure the tap trajectory passed straight though the thread hole. Fluoroscopy was used to verify the tap threads extended beyond the PSB apex. The measured screw was placed in the bone and manually tightened by the same surgeon using two fingers and the thumb on the screwdriver to a perceived maximal torque. This approach is used in clinical cases to avoid breaking the screw head or overtightening the screw leading to subsidence. A torque limiter was not used. For the CS_single_ group, an identical approach and procedure were used, except for using appropriately sized glide (4.5 mm) and thread (3.2 mm) holes and a conventional tap.

For the BSF_double_ or CS_double_ groups, the drill and implant sizes were the same as the BSF_single_ group. One screw was placed axial (laterally) while the other was placed abaxial (medially) to the mid‐point of the medial PSB base. Both glide holes were drilled, and the fracture reduced with bone reduction forceps prior to drilling the thread holes. The axial screw was placed first, followed by the abaxial screw. Once both screws were positioned, they were both manually tightened using the same maximal perceived torque stated above. Final placement was confirmed with radiography and fluoroscopy. Repaired constructs were wrapped in 0.9% saline‐soaked gauze and kept at −20°C until biomechanical testing.

### 2.3 Biomechanical testing

**FIGURE 2:**
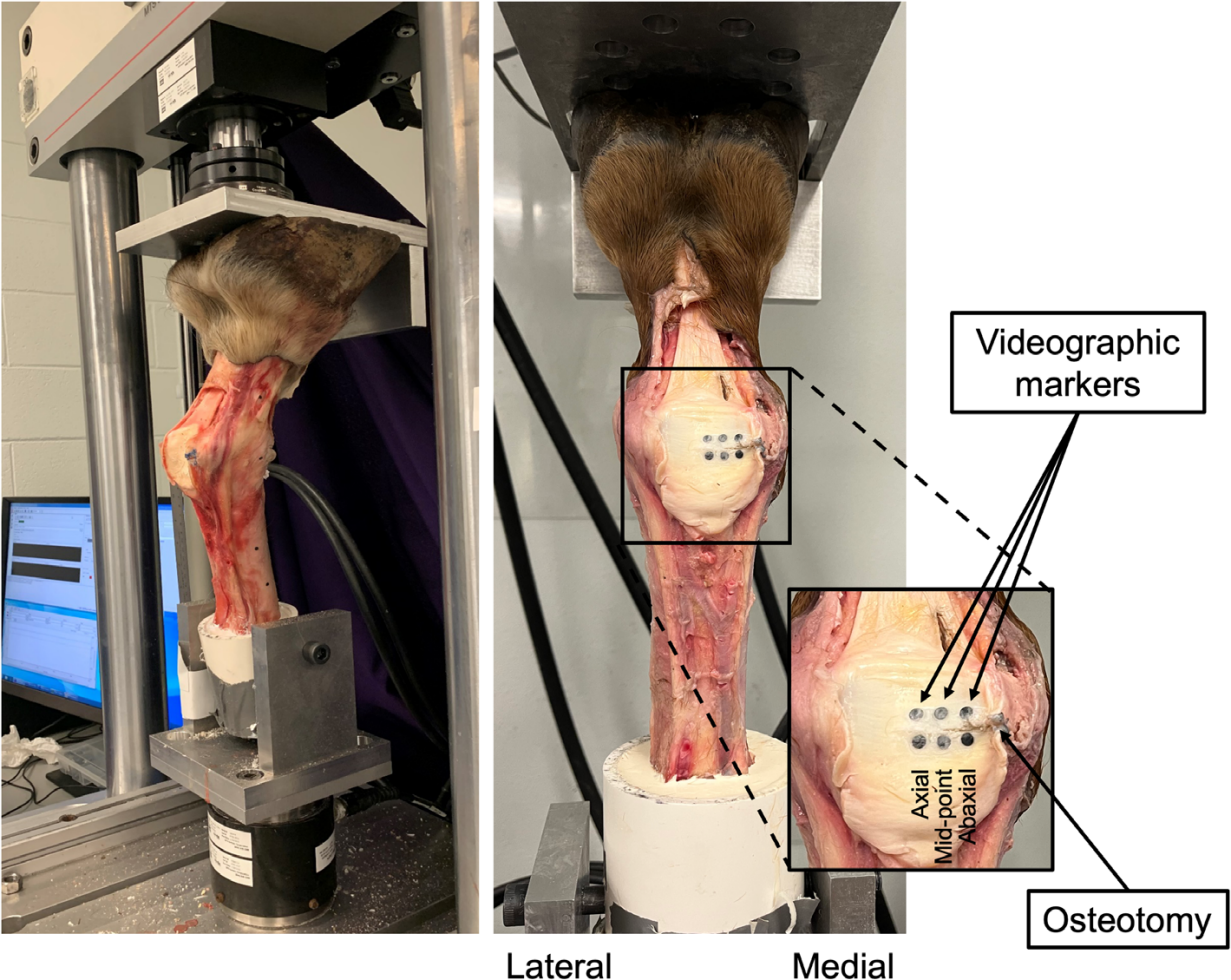
Biomechanical testing analysis setup showing limb positioning within the servohydraulic materials testing system where downward compression causes metacarpophalangeal joint extension (left). Right, palmar view of the setup showing placement of the videographic markers used to co‐register gap displacement with load‐deformation data.

Repaired constructs were allowed to thaw at room temperature (21°C) for 12 h prior to biomechanical testing. Two freeze–thaw cycles with total storage for less than 6 months for each specimen was used. To reduce stress concentration on the third metacarpal bone, the proximal aspect of each specimen was mounted in a polyvinyl chloride (PVC) sleeve (8.89 cm diameter, 1.27 cm thick, 10.16 cm long) using a strong two‐part hard casting resin (Smoothcast 321, Smooth‐On, Macungie, Pennsylvania). Constructs were suspended by the hoof, placed within the PVC sleeve so that the proximal aspect of the distal row of carpal bones was level with the top of the PVC sleeve. Once level within the PVC, the casting resin was poured into the PVC sleeve and allowed to set (approximately 15 min). Each limb was secured in a mechanical testing system (MTS, Model 805, MTS Corp., Eden Prairie, Minnesota) with a load cell (Model 661‐19‐01, Minimum load ‐5 kN, maximum load 5 kN) by drilling a 13 mm diameter hole perpendicular to the long axis of each specimen, through the PVC, casting resin and 3rd metacarpus approximately 2 cm distal to the carpometacarpal joint. A 12.5 mm diameter steel rod was inserted through the drill hole to attach each specimen to a servo‐hydraulic load frame which allowed axial loading of the limb (Figure 2). Three 3 mm diameter black markers were placed 1 mm proximal and distal to the osteotomy, at the abaxial, mid‐point and axial aspects of the osteotomy to allow for evaluation of gap displacement characteristics (Figure 2). The distance from the outside of the abaxial marker to the inside of the axial marker was measured using digital calipers (RS Pro 200 mm, RS, London, UK) and used as a reference scale to allow for measurements from digital images. A lateral to medial and palmar to dorsal radiographic projections (80 kV, 0.1 s, 15 mA, 50 cm focal distance) were taken prior to testing. The limb was secured in the MTS so that the long axis of the MC3 was aligned along the center of the longitudinal axis of the actuator. The hoof was secured in a custom design support, to allow for axial loading of the limb, and dorsal support of the hoof wall. Prior to testing, limbs were preloaded to normalize viscoelastic effects in which a static preload of 900 N was applied, immediately followed by testing.^13^ Constructs were tested in axial compression using a single displacement‐controlled cycle to failure at 4 cm/s until ultimate failure.^13^ Load and deformation data were recorded at 1000 Hz throughout each test and stored as a data file (Microsoft Excel, version 2307, Microsoft Corporation, Redmond, Washington).

### 2.4 Videography

All mechanical testing was recorded with digital videography (Nikon D3500, 60 frames/s, Nikon, TYO, Japan). For each specimen test, digital video recording was started in synchrony with the beginning of biomechanical testing. The time from the start of the test to ultimate failure was recorded directly from the biomechanical testing data output. Video images were then extracted using VLC Media Player (VLC Media Player, Video LAN, PAR, France), and time‐stamped according to the number of extracted images relative to test duration. Time‐stamped images were then verified against the load‐deformation data. Gap measurements were calculated for time‐stamped images using ImageJ software (National Institutes of Health, Bethesda, Maryland) corresponding to yield, construct failure and ultimate failure points.

### 2.5 Data collection

Subjective impressions of BSF or CS placement during surgical repair were documented informally. Time, load and displacement data were analyzed to generate load‐deformation curves for all mechanical tests (Microsoft Excel). Construct stiffness was calculated as the initial linear portion of the load‐deformation curve. The point of structural yield (failure of fracture repair) was defined as the first point where the curve deviated from the first linear region by a greater than 1% drop in load.^13^ Construct failure was determined using the load‐deformation curves as the first point after construct yield where a drop in load occurred resulting in a gap strain of zero, which was verified against time stamped images showing visible separation of the osteotomy (gap displacement). Prior to construct failure, there was confirmation that no visible gapping, strain or displacement occurred. Ultimate failure was defined as the point in which the construct was unable to withstand any further load (complete loss of structural integrity; zero load) and confirmed by correlating load‐deformation data with videographic images.

Gap failure characteristics were evaluated from time stamped videographic images, corresponding to the yield, construct failure and ultimate failure points. The distance between the abaxial, mid‐point and axial markers proximal and distal to the osteotomy were measured in triplicate by measuring three times on the same frame and the mean value was calculated for each location. At 900 N preload, the distance between markers was measured at the beginning of the trial and recorded as 0 mm displacement. Changes in displacement were calculated as total displacement relative to baseline, and recorded at the yield, construct failure and ultimate failure points. Modes of failure were recorded for each specimen and evaluated using videography that corresponded to ultimate failure on the load‐deformation curve, gross evaluation and comparison of pre‐ and post‐test radiographs. Proportions and characteristics of construct failure were evaluated for screw deformation, concurrent fractures, and soft tissue disruption and compared between groups.

### 2.6 Statistical analysis

Sample size calculations were determined using published data from Eddy et al. The mean ± SD load of a 4.5 mm cortical screw at yield was 1800 N (+/−300 N).^13^ Six paired limbs will enable the detection of a mean difference of 600 N between groups with a power of 0.9 with a type I error rate (critical alpha) of 0.05. Quantitative data are presented as mean ± SD. Outcome variables reported for all groups included the biomechanical outcome parameters (yield, construct failure, ultimate failure, construct stiffness) and fracture gap separation at the osteotomy. Statistical analysis was performed using GraphPad Prism (version 10.4.1, Boston, Massachusetts). Normality was assessed using the Shapiro–wilk test. Paired samples were compared using the Wilcoxon matched‐pairs signed rank test, and unpaired samples were compared using the Mann–Whitney test. Fisher's exact tests were used to compare failure mode proportions between groups. Statistical significance was defined at *p* < .05.

## 3 RESULTS

**FIGURE 3:**
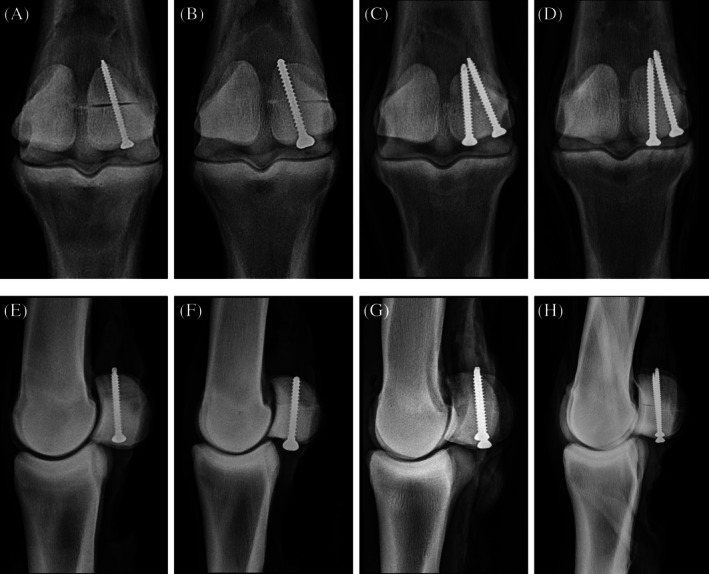
Representative dorsopalmar (A–D) and lateromedial (E–H) radiographic projections of each test group after repair of osteotomized medial proximal sesamoid bone fractures using (A, E) a single 3.5 mm bone screw fastener (BSF), (B, F) a single 4.5 mm cortex screw, (C, G) double BSF, and (D, H) double 3.5 mm cortex screws.

Seven repair constructs were completed per group as intended (28/28 total constructs) (Figure 3). To achieve this, a total of 32 limbs (from 16 horses) were ultimately collected to accommodate two replacement limbs (with their contralaterally matched control limbs). There was no observable damage to the suspensory branches, intersesamoidean ligament or distal sesamoidean ligaments during creation of the PSB osteotomy in any of the limbs. The medial aspect of the intersesamoidean ligament dorsal to the palmar fibrocartilage could not be directly viewed, though palpation with a probe after osteotomy did not reveal obvious damage in any limb. No implant placement issues were observed in the CS_single_ or CS_double_ groups. Insertion of the BSF tap and screws had subjectively greater insertional torque compared to CS. In one limb assigned to the BSF_single_ group, the threads sheared during screw insertion preventing compression. In one limb of the BSF_double_ group, the BSF tap broke while creating the abaxial thread hole. In both cases, the implanted and contralateral limbs were discarded and replaced (4 total) to ensure only correctly placed screws were assessed with biomechanical testing, which also maintained equivalent numbers per group. In all specimens repaired using two screws, the axial screw was generally the most challenging to place due to interference with the heel of the foot.

**FIGURE 4:**
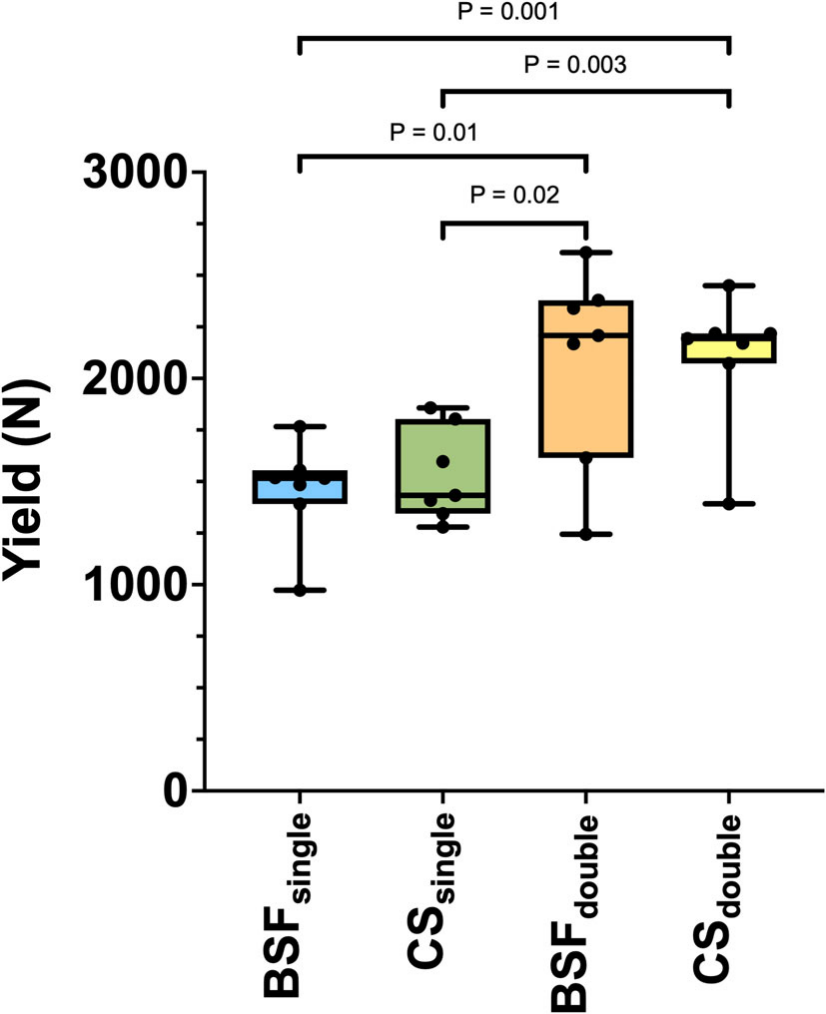
Box plots (median, interquartile range, minimum, maximum) showing yield failure of each treatment group used to repair a simulated medial proximal sesamoid fracture. BSF, bone screw fastener; CS, cortex screw.

There were no differences in construct stiffness between groups (Table 1). Specimens repaired in the BSF_double_ group (2081 ± 181 N) had significantly greater yield loads compared to the BSF_single_ (1458 ± 92 N, *p* = .01) and CS_single_ (1532 ± 86 N, *p* = .02) groups (Table 1). The CS_double_ group (2101 ± 126 N) had significantly higher yield loads compared to the BSF_single_ (*p* = .001) and CS_single_ (*p* = .003) groups (Figure 4, Table 1). There was a significantly higher construct failure load for specimens in the BSF_double_ group (3064 ± 764 N) compared to the CS_single_ group (2237 ± 206 N, *p* = .04, Table 1). There were no differences in yield, construct failure or ultimate failure loads between single screw repair groups (BSF_single_ vs. CS_single_ groups) or between double screw repair groups (BSF_double_ vs. CS_double_ groups) (Table 1).

**TABLE 1:** Biomechanical testing variables (mean ± SD) of different surgical repair methods of simulated equine medial mid‐body PSB fractures.

Group	Stiffness (*N*/mm)	Yield (*N*)	Construct failure (*N*)	Ultimate failure (*N*)
BSF_single_	97.5 ± 8.7	1458 ± 92^a^	2973 ± 483	6756 ± 721
CS_single_	101.7 ± 24.8	1532 ± 86^a^	2237 ± 206^a^	6975 ± 649
BSF_double_	95.6 ± 17.8	2081 ± 181^b^	3064 ± 764^b^	5591 ± 1475
CS_double_	99.0 ± 24.1	2101 ± 126^b^	2697 ± 700	5951 ± 1410

*Note*: Different superscript letters indicate differences between treatment groups within a column.

Abbreviations: BSF, bone screw fastener; CS, cortical screw; PSB, proximal sesamoid bone.

**FIGURE 5:**
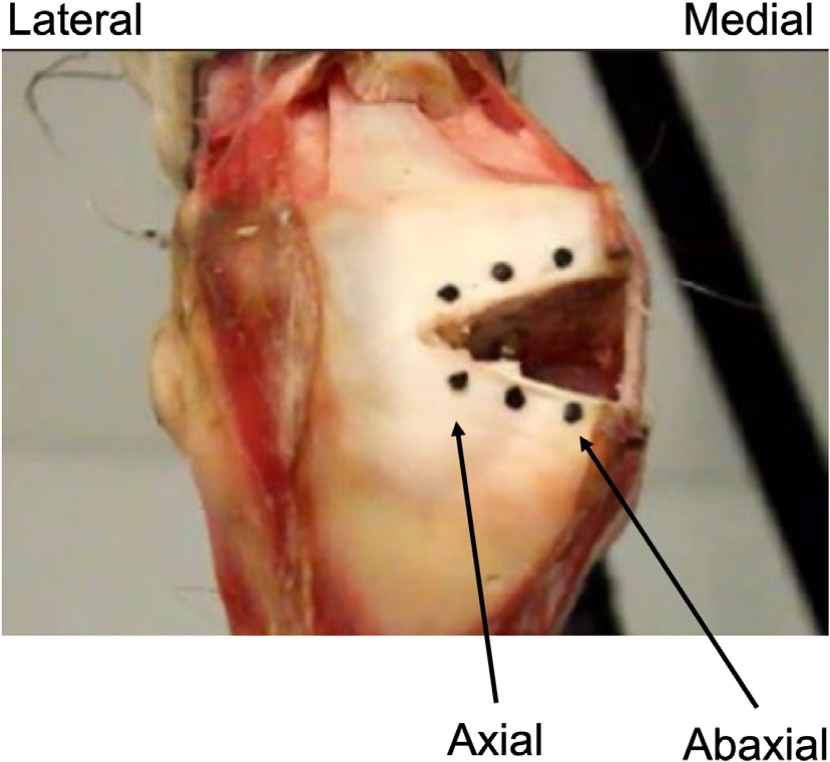
Photograph taken at construct failure after repair of a simulated medial mid‐body proximal sesamoid bone fracture showing higher gap displacement at the abaxial aspect of the osteotomy compared to the axial aspect. Proximal is to the bottom of the image.

In all groups at construct failure, there was significantly higher gap displacement abaxially on the medial PSB compared to axially (BSF_single_: *p* = .002, CS_single_: *p* = .001, BSF_double_: *p* = .015, CS_double_: *p* = .008) (Figure 5, Table 2, Video S1). There were also significant differences between abaxial and mid‐point markers (BSF_single_: *p* = .002, CS_single_: *p* = .003, BSF_double_: *p* = .026, CS_double_: *p* = .037), and between mid‐point and axial markers (BSF_single_: *p* = .003, CS_single_: *p* = .004, BSF_double_: *p* = .013, CS_double_: *p* = .013) within each construct group. There were no differences between repair groups within each marker location (abaxial, mid‐point or axial).

**TABLE 2:** Gap displacement (mean ± SD) of different surgical repair methods at the abaxial, middle and axial aspects of the osteotomy site at construct failure of simulated equine medial mid‐body PSB fractures.

Group	Gap displacement (mm)			Abaxial vs. axial	Abaxial vs. middle	Middle vs. axial
	Abaxial aspect	Middle aspect	Axial aspect	*p*‐value	*p*‐value	*p*‐value
BSF_single_	6.5 ± 3.2	4.7 ± 2.7	2.6 ± 1.9	.002	.002	.003
CS_single_	7.5 ± 3.3	5.0 ± 2.3	3.3 ± 2.5	.0006	.003	.004
BSF_double_	7.2 ± 3.4	4.4 ± 2.1	1.8 ± 1.6	.015	.026	.013
CS_double_	9.2 ± 1.2	7.9 ± 1.5	4.0 ± 2.1	.008	.037	.013

*Note*: *p*‐values indicate the significance level of comparisons between gap displacement between the abaxial, middle and axial aspects within the same group/row.

Abbreviations: BSF, bone screw fastener; CS, cortical screw; PSB, proximal sesamoid bone.

All groups demonstrated failure of repair in tension through the PSB, separating into two or more fragments, however additional failure features were observed, which varied between groups. Screw deformation was significantly associated with repair groups (*p* = .0006); it was observed in all the groups using 3.5 mm screws (BSF_single_: 7/7) while it was not observed in any within the 4.5 mm screw group (CS_single_: 0/7; Table 3). Deformation of the screws occurred by bending within the frontal and sagittal planes. When bending occurred, the convex aspect of the bent screws was principally at the palmar surface but also located medial (abaxial). Double screw repair with 3.5 mm screws had a lower prevalence of deformation (BSF_double_ & CS_double_ both 2/7) than single screw repair (BSF_single_:7/7). Deformations consisted of bending at the mid‐point of the screw at the level of the osteotomy. Concurrent apical fragmentation of the repaired PSB was common but not different between groups (BSF_single_:5/7, CS_single_:4/7, BSF_double_:6/7, CS_double_:6/7) (Table 3). Concurrent lateral PSB fracture was observed occasionally in all groups but was not different between groups (BSF_single_:3/7, CS_single_:4/7, BSF_double_:1/7, CS_double_:2/7) (Table 3). Soft tissue disruption within the medial suspensory branch (proximal to the osteotomy site) at construct failure was common, though was not different between groups (BSF_single_:4/7, CS_single_:4/7, BSF_double_:6/7, CS_double_:5/7) (Table 3).

**TABLE 3:** Features of failure (percent [proportion]) of different surgical repair methods at the abaxial and axial aspects of the osteotomy at construct failure of simulated equine medial mid‐body PSB fractures.

Group	Additional features of construct failure			
	Screw deformation*	Apical fragmentation	Concurrent lateral PSB fracture	Medial suspensory branch disruption
BSF_single_	100% (7/7)	71% (5/7)	43% (3/7)	57% (4/7)
CS_single_	0% (0/7)	57% (4/7)	57% (4/7)	57% (4/7)
BSF_double_	29% (2/7)	86% (6/7)	14% (1/7)	86% (6/7)
CS_double_	29% (2/7)	86% (6/7)	29% (2/7)	71% (5/7)

Abbreviations: BSF, bone screw fastener; CS, cortical screw; PSB, proximal sesamoid bone.

* Fisher's exact test revealed a significant overall association between screw deformation and repair group (*p* = .0006). Other features had no association with repair group.

## 4 DISCUSSION

This study showed insufficient evidence to detect a biomechanical difference between the BSF and CS implants in a simulated model of equine medial mid‐body PSB fracture repair. Biomechanically, both double screw repairs using two 3.5 mm screws (BSF_double_ or CS_double_) had higher yield values than single screws (BSF_single_ or CS_single_) in partial support of our hypothesis. Our other hypothesis that the BSF would have reduced gap formation or a higher yield than a 4.5 mm CS was not supported by these data. Despite its smaller size, a single 3.5 mm BSF screw was not different in yield, construct failure or ultimate failure when compared to a single 4.5 mm CS. Gap displacement in all repair groups showed that the abaxial surface of the medial PSB displaced more than axial locations irrespective of treatment group while under axial loading conditions.

There was insufficient evidence to detect a difference in biomechanical properties between the constructs repaired with the 3.5 mm BSF compared to the larger 4.5 mm CS under single axial loading conditions to failure. While the 4.5 mm cortical screw has a larger core diameter than the BSF (3.1 vs. 2.4 mm), the BSF was not available in a size greater than 3.5 mm during this study. A separate experimental group including a single 3.5 mm CS was considered, though due to a recent study directly comparing a 3.5 mm BSF to 3.5 mm CS^17^ and the lack of a single 3.5 mm screw being used clinically, this group was omitted. Comparison to a 4.5 mm cortical screw in this study design was used to replicate current clinical practice based on currently available screws.^9^ One possibility for the lack of a difference in biomechanical results between a 3.5 mm BSF and 4.5 mm CS may be attributed to the unique thread design of the BSF. Other studies investigating features of the BSF have shown that it creates an interlocking bone‐implant interface that distributes forces from the implant to the bone, thereby resisting loads in all directions.^15^, ^18^ This current study did not specifically investigate the bone‐screw interface. However, the BSF groups had no different yield than CS groups with the same number of screws, yet the BSF groups had higher construct failure. This biomechanical reversal between yield and construct failure could suggest that the interlocking threads may have some effect. Although in vivo forces are complex and multidirectional,^16^ the current study tested only uniaxial loading conditions. Under mid‐stance load, both PSBs experience external rotation with a variable degree of abduction.^16^ The BSF thread design functions to resist multidirectional loads within the PSB while buttress threads present in CSs are principally designed to resist uniaxial pullout forces.^18^ In a recent study, buttress threads were shown to have superior axial pullout strength in equine third metacarpal bone compared to the BSF.^17^ However, in that study, pullout force was unidirectional and direct extrapolation of those results to the equine PSB is unlikely to be accurate.

The higher yield in double screw repair compared to single repair is hypothesized to be due to improved force distribution and reduction of rotational instability. Once the construct deforms at yield, there is no elastic recoil to return it to its prior position, which with cyclical loading in vivo risks a fibrous union or progresses to construct failure. While the single 4.5 mm CS is larger and is less likely to deform, it is still unable to counteract rotational forces on the repaired PSB. Maximal compression of the fracture gap when using lag technique could make rotational forces less likely. However, during cyclic loading, micromotion fosters implant loosening leading to rotational instability. Double screw repairs are less susceptible to rotational forces and benefit by sharing load distributions between both screws,^21^ particularly in the equine PSB which has components of multi‐directional loading.^16^ While repairs using two differently sized screws within the same PSB was not performed due to lack of current clinical use, there may be advantages to considering this in the future.

Detection of higher yields despite similar stiffness between groups could occur due to a variety of reasons. It could be due to the constructs being compared having similar elastic behavior but different plastic thresholds, different design features of the screws, or the mode of failure versus the initial response. Stiffness measures the initial response of loading (relatively lower loads), while yield measures displacement at the onset of failure (relatively higher loads). Since the screws were composed of the same material, the differences are likely attributed to having different plastic thresholds or screw design factors. However, further work is necessary to corroborate those suspicions.

Repair of transverse, mid‐body PSB fractures is technically challenging. In this study, we found that placement of a single screw was less challenging than the placement of two screws. Insertion of the most axial screw often relied on the surgeon's hand and drilling equipment being much closer to the medial heel of the foot. In addition to using long drill bits, once the reduction forceps were placed onto the PSB, extension of the foot and fetlock helped make room for this instrumentation. This issue was prevalent in all limbs used, irrespective of breed and screw repair type. Compared with clinical cases, placement of the pointed reduction forceps and insertion of screws was likely easier due to the lack of skin and flexor tendons in this ex vivo study. However, the use of two 3.5 mm screws is performed by many equine orthopedic surgeons currently. Thus, the repair techniques used in this study are likely transferrable to clinical cases.

Insertion of the BSF tap and screws had subjectively greater insertional torque compared to CS. This property has been characterized for the BSF by others^17^, ^18^ and may have been a result of the interlocking threads interdigitating with bone. We observed that the threads of one BSF sheared during insertion as well as a broken tap. It is possible that the thread hole was incompletely tapped (though unlikely as fluoroscopic confirmation was used) or not aligned straight within the glide hole, which caused higher insertional torque. This slight eccentric placement could have caused thread shearing and a broken tap. Broken and bent taps are often encountered in equine bone due to its high stiffness,^22^ and similar issues with the tap may have occurred with the CS group if the group size was larger. We suspect the BSF system as a function of its multidirectional threads would conceivably be more susceptible to blunting and therefore breaking with repeated use. In this case, both situations occurred after a single tap was used repeatedly. Thread shearing may be overcome by placement of a new screw and could be considered a minor complication. In contrast, tap breakage, particularly in the equine PSB with limited bone to place another screw is considered a serious complication, particularly if it cannot be removed without collateral damage, thereby weakening the bone further.

To our knowledge, this is the first study to biomechanically evaluate transverse‐PSB fractures from the palmar aspect. All repair strategies demonstrated consistently greater gap displacement abaxially tested under single cycle to failure. The PSB not only experiences variable degrees of abaxial displacement and abduction, but high levels of tensile forces from the suspensory and distal sesamoidean ligaments during metacarpophalangeal joint extension.^23^ Increased abaxial displacement at the osteotomy suggests that tensile forces are greatest at this site. Microstructural evaluation of fractured equine PSBs show that a focal articular, and subchondral osteopenia exists at this abaxial mid‐body location.^24^, ^25^ The increased tensile forces at the site of focal subchondral weakness may initiate mid‐body PSB fracture. The relevance of greater abaxial displacement in vivo requires further investigation. While surrounding soft tissues such as the superficial and deep digital flexor tendons were removed for this study, they are located palmar and seemingly have less contribution to counteracting abaxial PSB forces. Their influence on load sharing around the fetlock requires further investigation. The convex portion of the bent screws being both palmar and medial suggest that both these PSB surfaces underwent tension during testing. While full characterization of compression and tension surfaces of the PSB were not investigated here, further studies are indicated to determine whether surgical repair should also attempt to neutralize abaxial forces in PSB repair and perhaps may explain why double screw repair has higher yield loads than single screw repair.

All specimens demonstrated failure at the osteotomy at single cycle to failure. Most specimens also demonstrated apical fragmentation, while screw deformation varied between constructs. Apical fragmentation is likely the result of increased holding power at the PSB base, the bone screw interface in the thread hole, and lower bone volume at the apex.^13^, ^14^ Interestingly, apical fragmentation consistently occurred at the abaxial aspect of the PSB apex. We speculate the direction of tensile forces acting on the PSB is the cause, although further studies are required to quantify the force magnitude and direction at these sites. All specimens repaired with a single BSF demonstrated deformation, whereas none of the 4.5 mm CSs exhibited deformation. In double screw repair with both BSF and CS, deformation occurred albeit less prevalent than a single 3.5 mm screw. Likely explanations are due to the larger area moment of inertia in the 4.5 mm screw, which resists greater bending forces, while double screw constructs distribute forces more evenly across the fracture.

Translating the results of this biomechanical study to in vivo situations is not simple. Single load to failure at slower loading rates was needed to capture the mechanical and videographic data, similar to other studies.^12^, ^13^ Additionally, only soft tissue structures with direct attachment to the PSB were included. In vivo cyclic and fast loading rates along with the influence of surrounding soft tissue structures (e.g., flexor tendons) and bone healing measures cannot be easily replicated ex vivo. Cyclic testing in cadaveric loading studies should be considered in future studies, though still does not represent the true in vivo environment and physiologic response to external loading. Micromotion and testing of the multidirectional benefits of the BSF as well as contribution of surrounding soft tissues are therefore best determined in vivo or in repeating biomechanical studies with full musculotendinous units intact. Clinically, PSB repair is often combined with cast placement. We did not include this potential source of variation as our objectives were to scrutinize different screw configurations for PSB repair and not all factors that contribute to stability of the distal limb. While more clinical data are needed, BSFs have been successfully used for mid‐body PSB fracture repair without complication in clinical cases (R. S. Carpenter unpublished data).

Creation of this simulated fracture model required transection of the palmar fibrocartilage over the medial PSB and the influence of transection on higher abaxial gap formation is unknown. The amount of fibrocartilage damage in clinical cases of PSB fracture is not described and is likely due to its position within the digital flexor tendon sheath, while arthroscopic view of the fetlock is most critical to accurately re‐align the articular surface of the fractured PSB.^9^ Based on thin palmar fibrocartilage and its integration with the palmar surface of the PSBs, there could have been variation in weakening the fibrocartilage during osteotomy if left intact. Thus, transection of the fibrocartilage enabled osteotomy consistency, ability to track gap formation and if anything put higher strain on the repair than would be experienced in vivo. Limbs of varying breeds were used in this study due to our hospital case population and could have led to data variability. However, we did not observe any clear differences between breeds. Despite the same surgeon tightening all screws using the same two‐finger method, there may be variation in screw tightness/fracture compression. Alignment in potting was done visually and could be a source of biomechanical variation. PSB integrity was assessed visually and with radiographs: we did not evaluate the microstructural characteristics or bone volume fractions pre‐emptively. While the second screw was placed abaxially due to available PSB bone area, which seems to be ideal, other configurations including optimal screw spacing were not attempted. Further studies are required to determine whether this positioning of two screws for repair of transverse mid‐body PSB fractures is optimal.

The single 3.5 mm BSF used to repair a simulated mid‐body PSB fracture was not biomechanically different in gap displacement or yield from a 4.5 mm CS under single cycle to failure. Screw deformation occurred in a higher proportion in the single BSF group at construct failure. Use of two screws (either BSF or CS) had higher yield to failure than single screw repairs. Biomechanical properties of BSF implants were not found to be different compared to CSs. Determination of the effect of the multidirectional features of the BSF threads requires additional investigation in vivo. Regardless, higher gap displacement on the abaxial portion of the PSB suggests any repair strategies should incorporate abaxially placed implants where possible, though further investigation in vivo is warranted.

## AUTHOR CONTRIBUTIONS

O'Brien TJ, BVSc, MS, DACVS (Large Animal): Provided substantial contribution to the study conception and design, performed surgical repair, acquired, analyzed and interpreted the data, and drafted the manuscript. Johnson JW, BS, PhD: Provided contributions to biomechanical assessments, acquired and interpreted data and critically revised the manuscript. Kawcak CE, DVM, PhD, DACVS: Contributed to study conception and design, interpreted data and critically revised the manuscript. Gadomski BC, BS, PhD: Contributed to biomechanical assessments, interpretation of data and critically revised the manuscript. Carpenter RS, DVM, MS, DACVS (Large Animal): Provided contribution to study design, interpretation of data and critically revised the manuscript. Nelson BB, DVM, PhD, DACVS (Large Animal): Provided substantial contribution to study conception and design, supervised surgical repair, analyzed and interpreted data and critically revised the manuscript. All authors approved of the final version and agree to be accountable for all aspects of the work.

## CONFLICT OF INTEREST STATEMENT

The authors have no conflicts of interest to disclose.

## Supporting information


**Video S1.** Mechanical testing video demonstrating higher abaxial displacement.
